# Tumor conspicuity significantly correlates with postoperative recurrence in patients with pancreatic cancer: a retrospective observational study

**DOI:** 10.1186/s40644-020-00321-2

**Published:** 2020-07-10

**Authors:** Hye Jin Yoo, Myung-Won You, Dong Yoon Han, Ji Hye Hwang, Seong Jin Park

**Affiliations:** Department of Radiology, Kyung Hee University Hospital, Kyung Hee University College of Medicine, 23, Kyungheedae-ro, Dongdaemun-gu, Seoul, 02447 Republic of Korea

**Keywords:** Pancreatic cancer, Computed tomography, Recurrence, Prognosis, Prognostic factors

## Abstract

**Background:**

There has been scanty data regarding the clinical significance of tumor conspicuity in pancreatic cancer. In this study, we attempted to investigate the prognostic significance of pancreatic tumor conspicuity and determine prognostic factors for postoperative recurrence in patients with surgically resected pancreatic cancer.

**Methods:**

Between January 2011 and September 2019, 62 patients who underwent preoperative computed tomography (CT) for pancreatic cancer were retrospectively included. Two reviewers evaluated various clinical, imaging, and pathologic variables and reviewed all available medical records to determine patient outcomes after surgery. Tumor conspicuity was defined as the attenuation ratio between normal parenchyma and tumor lesions on dynamic-enhanced CT images and represented the conspicuity score. Recurrence-free survival and overall survival were investigated using Cox regression analysis.

**Results:**

Patient mean age was 65.9 (±11.6) years, and 56.5% were male. The median follow-up period was 11 months (range 2–138). Forty patients (64.5%) experienced postoperative recurrence, and the median time to recurrence was 6 months (range 1–101). Tumor conspicuity scores were positively correlated with both radiologic and pathologic tumor sizes (r = 0.252, 0.321, *p* < 0.01). Conspicuity score ≥ 2 (HR 3.8, 95% CI 1.73–8.47), elevated preoperative (HR 1.15, 95% CI; 1.02–1.28) and postoperative CA19–9 (HR 1.11, 95% CI 1.01–1.23), pathologic tumor size (HR 1.61, 95% CI 1.06–2.45), and lymphatic invasion (HR 2.76, 95% CI 1.22–6.21) were significant factors for recurrence-free survival in the multivariate analysis.

**Conclusions:**

Over half of the patients with pancreatic cancer experienced postoperative recurrence (64.5%). Increased tumor conspicuity correlated with larger tumor size and postoperative recurrence.

## Introduction

Pancreatic ductal adenocarcinoma (PDAC) is the third leading cause of cancer-related death worldwide, with a 5-year overall survival of 6–7.2% when all stages are considered [1, 2]. Surgery with adjuvant chemotherapy offers the best chance of disease cure; however, only 10–20% of patients present with resectable disease, and even after resection, the cancer recurs in up to 80% of patients, mostly within 2 years after surgery [3]. This high recurrence and dismal prognosis have been attributed to the presence of occult micrometastatic disease at the time of resection and lack of effective systemic therapies [4]. Based on current trends, it is anticipated that PDAC will become the second leading cause of cancer-related death by 2030 [5].

Pancreatic cancer is heterogeneous in clinicopathologic and imaging features, and not all cancers have the same biologic behaviors. Stage of tumor-node-metastasis (TNM), carbohydrate antigen 19–9 (CA 19–9) level, and tumor differentiation are known prognostic factors for patients with pancreatic cancer [6].

In contrast-enhanced computed tomography (CT) or magnetic resonance imaging (MRI), PDAC typically manifests as a hypovascular, low-signal intensity mass or nodule compared with the pancreatic parenchyma [7]. However, there is a small subset of visually isoattenuating PDAC in which tumor attenuation on contrast-enhanced CT is indistinguishable from attenuation of the pancreatic parenchyma and has characteristic clinical and pathologic features [8]. The isoattenuating PDAC has a more favorable postsurgical outcome that is associated with a high prevalence of well-differentiated tumors and R0 resections [9]. Dynamic MRI can detect 80% of isoattenuating PDAC; however, treatment decision is made in accordance with the resectability status based on CT findings. Further, there is a lack of data on tumor conspicuity at CT and its prognostic significance in PDAC patients.

Therefore, the aim of this study was to investigate the prognostic significance of tumor conspicuity determined by contrast-enhanced CT images and to identify prognostic factors for postoperative recurrence in patients with surgically resected pancreatic cancers.

## Materials and methods

### Patients

**Fig. 1:**
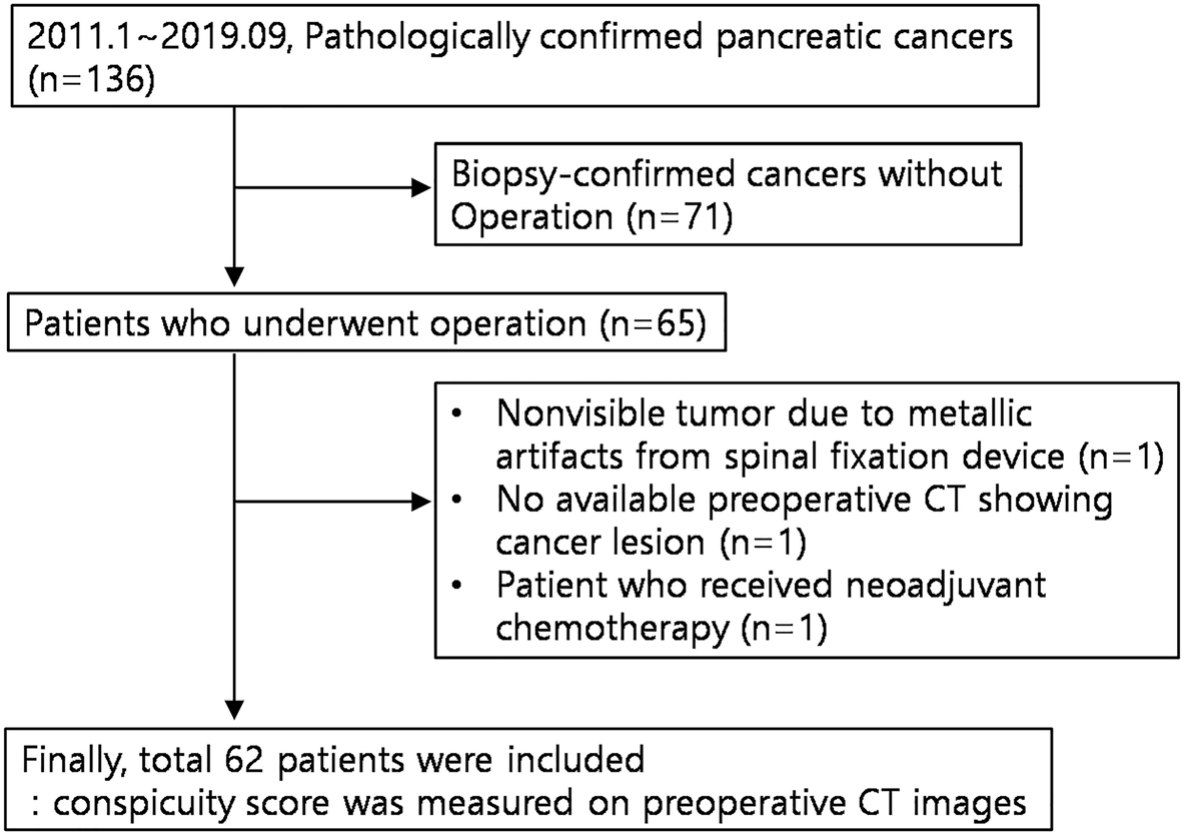
Flowchart of patient selection

Our Institutional Review Board approved this retrospective study, and the requirement for informed consent was waived. Between January 2011 and September 2019, 136 consecutive patients with pathologically confirmed pancreatic cancer were identified at our institution. Among these patients, 72 who underwent biopsy without operation (*n* = 71), one with a tumor that was not visible on CT images due to metallic artifacts (*n* = 1), and one who had no available preoperative CT showing their cancer lesion (n = 1) were excluded. This patient underwent initial CT scan 10 months prior to operation, but no cancer lesion was delineated in the pancreas. Another patient who received neoadjuvant chemotherapy was also excluded because tumor conspicuity on preoperative CT might have been altered by chemotherapy. Therefore, a total of 62 patients was included in this study. We reviewed all available medical records and imaging studies for these patients and evaluated clinical, imaging, and pathologic data and patient outcomes (Fig. 1).

### Clinical endpoints

The primary endpoint was recurrence-free survival, and the secondary endpoint was overall survival. Postoperative recurrence was determined by either 1) apparent local recurrence or/and distant metastasis in postoperative imaging studies (new soft tissue lesion, seeding nodule, liver metastasis, etc.), 2) suspected local recurrence or/and distant metastasis in postoperative imaging and subsequent progression with or without adjuvant chemotherapy, and 3) pathologically confirmed recurrence. Recurrence-free survival was defined as the time from operation to the first appearance of recurrence of pancreatic cancer on imaging studies. Secondary or double primary cancer was not considered as an event when calculating recurrence-free survival. Overall survival was defined as the time from the first visit or diagnosis of pancreatic cancer to the last visit or death. In the case of follow-up loss, the endpoint of follow-up time was the last visit just before the transfer or occurrence of follow-up loss. The median follow-up period was 11 months (range, 2–138).

### CT techniques

CT examinations were performed using one of the following multidetector CT scanners: 16-channel Lightspeed from GE Healthcare (*n* = 14), 64-channel Brilliance (*n* = 12), 128-channel Ingenuity (*n* = 28) from Philips Healthcare, and 64-channel Aquilion Toshiba (*n* = 6). The scanning parameters were as follows: a peak voltage of 120 kVp; a tube current-time product of 150–200 mAs with automated tube current modulation; a 3-mm slice thickness with a 3-mm reconstruction interval; a field of view of 300–380 mm; a gantry rotation time of 0.5–0.6 s; detector configuration of 0.625 mm; z-axis coverage of 24, 40, and 40 mm; pitch 0.9, 0.7, and 0.8 s; table speeds of 43.2, 47.5, and 63.8 mm per second; and a single breath-hold helical acquisition time of 9–10 s for 16-, 64-, and 128-channel CT exams. A total of 1.8–2.0 mL of nonionic contrast media (iohexol 350, Central Medical Service Co. Ltd.) per kilogram of body weight was injected at a rate of 3.1–3.2 mL/s with a 20-mL flush of normal saline following contrast injection. The scanning delay for arterial phase was 9–10 s after achieving enhancement of the descending aorta up to 200 HU. Following contrast media injection, arterial phase was performed at 30–35 s, portal phase at 70 s, and delayed phase at 3 min.

### Image analysis and quantitative measurement of tumor conspicuity: pancreas/tumor attenuation ratio (P/T AR) or T/P AR, represented by ‘conspicuity score’

Two radiologists with 11 and 4 years of clinical experience reviewed all the CT images in consensus. When there were disagreements between the two reviewers, a third reviewer with 26 years of clinical experience was consulted to resolve the disagreement. The reviewers were blinded to the pathologic results and patient clinical outcomes. Most of the patients underwent surgery within one month after initial imaging (55/64, 85%); however, 8 patients underwent delayed surgery more than one month after initial imaging. In these patients, image analyses were performed with preoperative imaging studies within at least 6 month before the operation. The following imaging features were analyzed: tumor location (head, body, and tail); tumor size (longest diameter in the axial plane); vascular invasion of the arterial system (celiac trunk, superior mesenteric artery, and common hepatic artery, etc.), venous system (main portal vein, portomesenteric confluence, superior mesenteric vein, etc.), or other (left renal vein, inferior vena cava, etc.); presence of obstructive pancreatitis; and resectability. Resectability was determined according to the National Comprehensive Cancer Network guidelines [10, 11]. We developed an imaging parameter to determine tumor conspicuity quantitatively: parenchyma/tumor (P/T) attenuation ratio (AR). Before measuring P/T AR, portal phase images were selected for tumor conspicuity measurement, and the three radiologists agreed on the location of tumors because portal phase was available in all the included cases. Within one week from this training session, the trainee radiologist with 4 years of experience drew a region of interest (ROI) on both the tumor and normal parenchyma to include at least two-thirds of the area of interest but avoiding necrosis, cysts, vessels, or calcifications, while maintaining areas of at least 20 mm^2^.Then the radiologist recorded the mean value of the ROI sizes and average signal intensities for tumor and parenchyma after repeating the measurements three times and calculated the AR for parenchyma and tumor. In cases where the attenuation of the tumor was larger than that of the parenchyma, the P/T AR was calculated as tumor/pancreas (T/P) AR, which is the inverse of P/T AR, to allow all ratios to be larger than 1. The P/T AR and inverse P/T AR (T/P AR) were collectively defined as the ‘conspicuity score’ and evaluated.

### Statistical analysis

Continuous variables were compared using Student’s t-test, and categorical variables were compared using Chi-square or Fisher’s exact tests between postoperative recurrence and non-recurrence groups. To determine prognostic factors for postoperative recurrence and death, univariate and multivariate Cox regression analyses for recurrence-free survival and overall survival were performed. Multivariate analysis was conducted for the significant variables on univariate analysis with a *p*-value cut-off less than 0.1 with adjustment for baseline characteristics including age and sex. The results are presented as hazard ratios with 95% confidence intervals. The survival curves were generated using the Kaplan-Meier method and compared using the log-rank test. All statistical analyses were performed using SPSS version 25 for Windows (SPSS Inc.). The significance level was set at *p* < 0.05 (two-tailed).

## Results

### Characteristics of the study population: clinical, imaging, and pathologic data

Tables 1 and 2 summarize the clinical, imaging, and pathologic data of the patients. The median preoperative and postoperative CA19–9 were higher than normal (133.1 and 34.4, respectively). Mean radiologic tumor size was 2.8 ± 0.8 cm, whereas pathologic tumor size was 3.2 ± 1.1 cm. Vascular invasion was present in 61.3% and major vascular invasion of the arterial and venous system in 37.1% (23/62) of patients. Most of the patient tumors were resectable or borderline resectable (96.7%), though two patients were classified as unresectable. The median ROI area in the normal parenchyma was 59.4 (range 22.72–121.12), and that in the tumor was 59.72 (range 23.9–127.9). The mean conspicuity score was 1.95 ± 0.85 (range 1.01–6.01). We set the mean conspicuity score of 2 as the cut-off value for dividing patients into subgroups. A conspicuity score < 2 was the low lesion contrast group, and a conspicuity score ≥ 2 was the high lesion contrast group; a conspicuity score ≥ 2 was seen in 40.3% (25/62) of patients. The majority of patients had moderately differentiated cancers (67.2%, 41/62) and T2 or T3 cancers (93.6%, 58/62). More than half of the patients were lymph node positive (61.3%, 38/62), and the majority of patients was TNM stage II (83.9%, 52/62). The resection margin was tumor positive in 17.7% of patients (11/62).

**Table 1:** Clinical and imaging characteristics of included patients (*N* = 62)

	N = 62
Age (yrs, mean ± SD)	65.97(±11.66)
Male (n,%)	35 (56.5)
Presence of diabetes mellitus (DM)	24 (38.7)
Charlson age-comorbidity index (CCI, median, range)	5 (2–9)
Preoperative CA19–9 (U/mL, median, range)	133.1 (2–12,000)
Postoperative CA19–9 (U/mL, median, range)	34.4 (0.39–12,000)
Interval between initial imaging and operation date (mo., median, range)	0.5 (0.5–20)
Adjuvant chemotherapy (n,%)	46 (74.2)
Location of tumor	
Head/ Body/Tail	38/12/12
Vascular invasion	38 (61.3)
Celiac trunk/SMA/CHA	7 (11.3)
PV/SMV/portomesenteric confluence	16 (25.8)
Others: IVC, left renal vein	3 (4.8)
Conspicuity score > 2	25 (40.3)
Radiologic tumor size (cm)	2.78(±0.87)
Resectability	
Resectable/borderline resectable/unresectable	37/23/2
Presence of obstructive pancreatitis (n, %)	50 (80.6)
Presence of double primary cancer (n,%)	6 (9.6)

*SD* standard deviation, *SMA* superior mesenteric artery, *SMV* superior mesenteric vein, *CHA* common hepatic artery, *PV* portal vein, *IVC* inferior vena cava

**Table 2:** Pathologic findings of included patients (*N* = 62)

	N = 62
Pathologic size (cm, mean ± standard deviation)	3.28(±1.12)
Tumor differentiation	
Well/moderate/poor	9/41/11
Positive resection margin (n,%)	11 (17.7)
Presence of microvascular invasion (n,%)	15 (25.4)
Presence of lymphatic invasion (n,%)	30 (50)
Presence of perineural invasion (n,%)	36 (65.5)
Pathologic T stage	
T1/T2/T3/T4	3/22/36/1
Pathologic N stage	
N0/N1/N2	24/30/8
TNM stage	
Stage I/II/III/IV	6/52/1/3
Presence of concomitant pancreatitis (n,%)	17 (27.4)

### Correlation of conspicuity score with tumor size

**Fig. 2:**
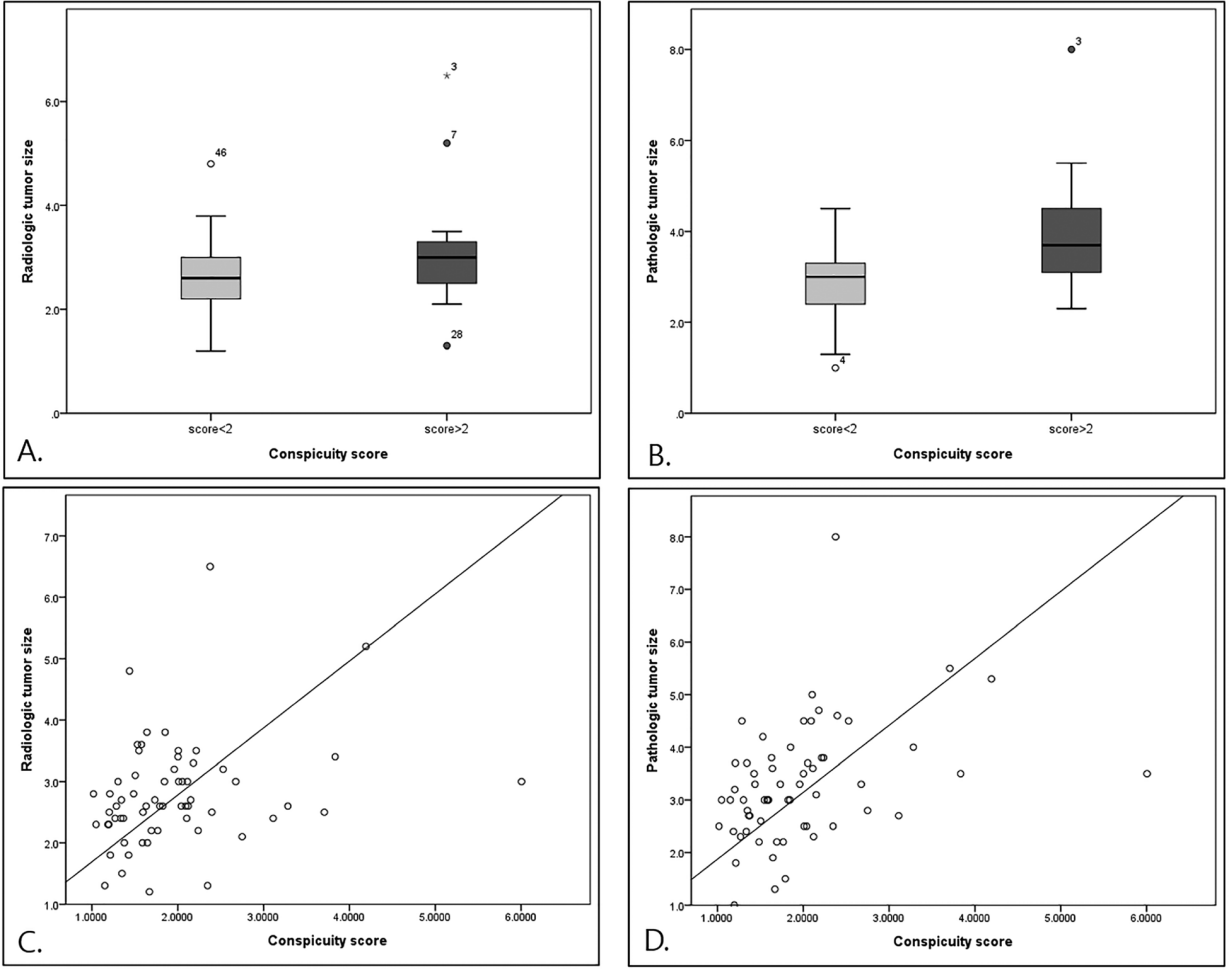
Correlation of conspicuity score with radiologic and pathologic tumor size A and B. Conspicuity score > 2 group shows larger tumor size in both radiologic (**a**, 2.6 ± 0.73 vs. 3.0 ± 1.01, *p* = 0.07) and pathologic (**b**, 2.8 ± 0.13 vs. 3.9 ± 1.25, *p* = 0.001>) exams, although there was no statistically significant difference in radiologic tumor size. C and D. Positive correlation between radiologic (**c**, r = 0.261, *p* = 0.041 and pathologic (**d**, r = 0.37, *p* = 0.003) tumor size and conspicuity score.

Figure 2 demonstrates the relationship between tumor size and tumor conspicuity. Both mean radiologic (2.6 ± 0.73 vs. 3.0 ± 1.01) and pathologic tumor sizes (2.8 ± 0.13 vs. 3.9 ± 1.25) were larger in the conspicuity score ≥ 2 (high lesion contrast) group than the conspicuity score < 2 (low lesion contrast) group. The conspicuity score ≥ 2 group showed significantly larger tumor size in pathologic exam (*p* < 0.001). When we examined the relationship between tumor size and conspicuity score continuous spectrum, there was a positive correlation between both radiologic (r = 0.261, *p* = 0.041) and pathologic tumor sizes (r = 0.37, *p* = 0.003) and conspicuity score. These results indicate that larger tumors had higher lesion contrast, in other words, increased tumor conspicuity.

### Survival data: univariate and multivariate analyses

**Fig. 3:**
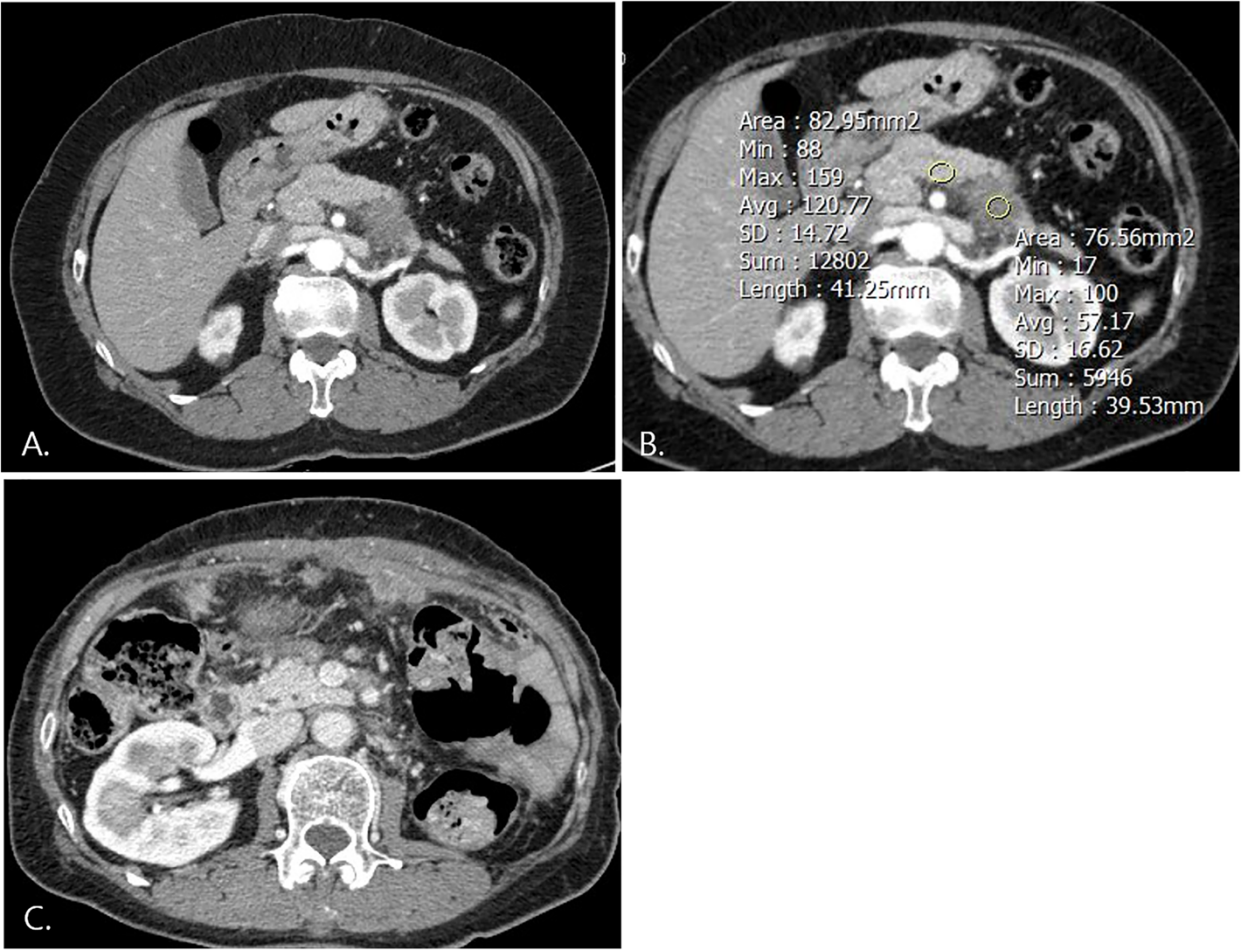
A 61-year-old female with pancreatic cancer: conspicuity score (P/T AR) ≥ 2. **a**. Approximately 3-cm-sized hypovascular mass in the pancreatic tail is clearly visible on arterial phase contrast enhanced CT. **b**. Conspicuity score was 2.11, preoperative CA 19–9 was 39.28, and postoperative CA19–9 was 35.44. The tumor was considered borderline resectable due to abutment to left renal vessels. **c**. Early recurrence occurred at 3 months after surgery, and peritoneal seeding progressed despite adjuvant chemotherapy. She stopped chemotherapy and transferred to an outside hospital for supportive care

**Fig. 4:**
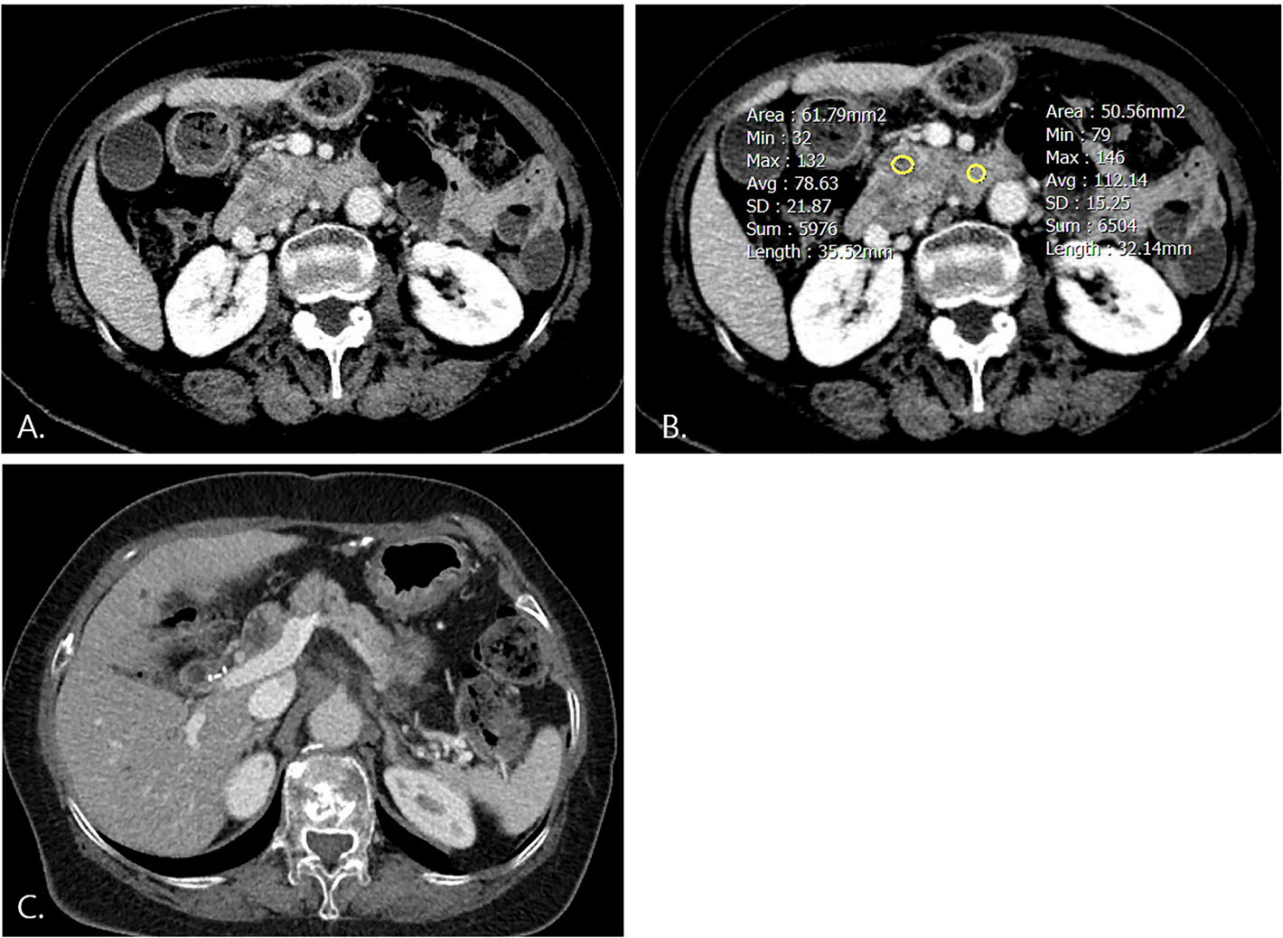
A 74-year-old female with resectable pancreatic cancer: conspicuity score < 2. **a**. Approximately 2.7-cm-sized hypovascular mass with low lesion contrast was visible in the pancreatic head on arterial phase contrast-enhanced CT. **b**. Conspicuity score was 1.42, preoperative CA 19–9 was 1466.33, and postoperative CA 19–9 was 7.88. **c**. There was no postoperative recurrence, and she is alive at 23 months after operation

Tables 3 and 4 show the survival data and patient outcomes. The median time to recurrence was 6 months (1–101), and more than half of the patients experienced postoperative recurrence (64.5%, 40/62). The mean duration for recurrence was 6.54 ± 8.27 (range, 0–37) months. Seven patients died, and 22 patients with follow-up loss were evaluated as alive until the last visit. Among the 40 patients with cancer recurrence, early recurrence within 12 months after surgery (80%) and distant metastases (80%, 32/40) developed in the majority. In univariate analysis for recurrence-free survival, adjuvant chemotherapy, preoperative and postoperative CA19–9, interval between initial imaging and surgery, unresectability, conspicuity score ≥ 2, pathologic tumor size, and lymphatic invasion were selected for further multivariate analysis due to *p*-values less than 0.1. Subsequent multivariate analysis showed preoperative (HR 1.15; 95% CI 1.02–1.29) and postoperative CA19–9 level (HR 1.11; 95% CI 1.01–1.23), conspicuity score ≥ 2 (HR3.67;95% CI 1.73–7.76), pathologic tumor size (HR 1.61; 95% CI 1.06–2.45), and presence of lymphatic invasion (HR 2.76; 95% CI 1.22–6.21) to be significant predictors for postoperative recurrence (Figs. 3 and 4). Table 5 shows the survival data of 54 patients after excluding eight patients who underwent delayed surgery at more than 1 month after initial CT imaging. Postoperative CA19–9 (HR 1.15;95% CI 1.02–1.28), pathologic tumor size (HR 2.06;95% CI 1.26–3.37), conspicuity score > 2 (HR 3.94;95% CI 1.74–8.95), and lymphatic invasion (HR 2.97;95% CI 1.15–7.63) were significant predictors for postoperative recurrence.

**Table 3:** Survival data for included patients (N = 62)

	N = 62
Postoperative recurrence (n,%)	40 (64.5)
Time to recurrence (mo., median, range)	6 (1–101)
Type of recurrence (n,%)	
Local/distant/combined	8 (20)/20 (50)/12 (30)
^a^Time of recurrence (n,%)	
Early/ late	32 (80)/8 (20)
Outcome of patients	
Alive/death	55/7
Median survival period (mo., median, range)	11 (2–138)

^a^Cut-off threshold is 12 months for differentiating between early and late recurrence

**Table 4:** Cox proportional regression analysis for postoperative recurrence (n = 62)

	Univariate: HR (95% CI)	*p*-value	^a^Multivariate: HR (95% CI)	*p*-value
Age	1.019 (0.989,1.050)	0.210	AD.	
Male	0.893 (0.474,1.684)	0.727	AD.	
Tumor differentiation			AD.	
Well				
Moderate	1.031 (0.419,2.535)	0.948		
Poor	1.187 (0.393,3.581)	0.761		
Interval between initial imaging and operation (mo.)	1.085 (0.990,1.189)	0.08	AD.	
CCI	1.044 (0.865,1.260)	0.653	0.918 (0.675–1.248)	0.585
Adjuvant chemotherapy	0.474 (0.242,0.926)	0.029*	0.544 (0.220–1.343)	0.187
Preoperative CA19–9	1.105 (1.007,1.214)	0.036*	**1.146 (1.022–1.286)**	0.02*
Postoperative CA19–9	1.105 (1.021,1.196)	0.014*	**1.114 (1.006–1.232)**	0.037*
Radiologic tumor size	1.248 (0.882,1.767)	0.211	1.483 (0.966–2.276)	0.072
Resectability				
Resectable	1		1	
Borderline resectable	1.560 (0.799,3.049)	0.193	1.868 (0.833–4.188)	0.129
Unresectable	4.018 (0.896,18.012)	0.069	1.349 (0.093–19.548)	0.826
Conspicuity score > 2	3.122 (1.616,6.029)	0.001*	**3.669 (1.735,7.762)**	0.001*
Pathologic tumor size (cm)	1.383 (1.058, 1.807)	0.018*	**1.612 (1.062–2.448)**	0.025*
Positive resection margin	1.528 (0.664,3.514)	0.318	1.407 (0.552–3.583)	0.475
Lymphatic invasion	2.058 (1.043,4.059)	0.037*	**2.757 (1.224–6.211)**	0.014*
Perineural invasion	1.659 (0.815,3.378)	0.163	2.448 (1.048–5.721)	0.039
Microvascular invasion	1.318 (0.61,2.848)	0.482	1.464 (0.644–3.327)	0.363
Pathologic T stage				
T1	1		1	
T2	0.417 (0.089,1.947)	0.266	0.583 (0.069–4.928)	0.620
T3	0.588 (0.135,2.588)	0.480	0.739 (0.09–6.055)	0.778
T4	0.402 (0.035,4.645)	0.465	0.535 (0.022–13.170)	0.702
Positive lymph node	1.178 (0.615, 2.256)	0.622	1.261 (0.605–2.630)	0.536

^a^ Adjusted for age, sex, concomitant pancreatitis, tumor location, tumor differentiation, and interval between initial imaging and operation

*HR* hazard ratio, *CI* confidence interval, *CCI* Charlson co-morbidity index

* *p-*value less than 0.05

**Table 5:** Cox proportional regression analysis for postoperative recurrence in 54 patients after excluding eight patients who underwent delayed surgery (*n* = 54)

	Univariate: HR (95% CI)	*p*-value	^a^Multivariate: HR (95% CI)	*p-value*
Age	1.028 (0.993,1.063)	0.117	AD.	
Male	0.822 (0.406,1.666)	0.587	AD.	
Tumor differentiation			AD.	
Well				
Moderate	1.090 (0.367,3.240)	0.877		
Poor	1.374 (0.378,4.998)	0.629		
Interval from initial imaging and operation (mo.)	0.934 (0.195,4.472)	0.932	AD.	
Charlson co-morbidity index	1.043 (0.847,1.286)	0.691	0.784 (0.526,1.170)	0.233
Adjuvant chemotherapy	0.39 (0.185, 0.821)	0.013*	0.393 (0.137,1.128)	0.393
Preoperative CA19–9	1.09 (0.979,1.213)	0.115	1.127 (0.984,1.291)	0.085
Postoperative CA19–9	1.112 (1.018,1.214)	0.019*	**1.148 (1.025,1.285)**	0.017*
Radiologic tumor size	1.097 (0.664,1.812)	0.719	1.409 (0.768, 2.585)	0.268
Resectability				
Resectable	1		1	
Borderline resectable	1.683 (0.806,3.515)	0.166	2.123 (0.876,5.144)	0.096
Conspicuity score > 2	3.173 (1.522,6.613)	0.002*	**3.944 (1.739, 8.945)**	0.001*
Pathologic tumor size (cm)	1.586 (1.026,2.452)	0.038*	**2.064 (1.265, 3.366)**	0.004*
Positive resection margin	1.32 (0.499,3.490)	0.575	1.147 (0.384, 3.427)	0.806
Lymphatic invasion	2.299 (1.048,5.045)	0.038*	**2.966 (1.153,7.629)**	0.024*
Perineural invasion	2.014 (0.818,4.958)	0.128	**3.569 (1.194,10.67)**	0.023*
Microvascular invasion	1.135 (0.454, 2.835)	0.787	1.545 (0.531,4.498)	0.425
Pathologic T stage				
T1	1		1	
T2	0.383 (0.079,1.846)	0.232	0.536 (0.062,5.124)	0.610
T3	0.600 (0.135,2.663)	0.502	0.918 (0.108,7.816)	0.937
Positive lymph node	1.157 (0.559, 2.392)	0.695	1.117 (0.47,2.653)	0.802

^a^ Adjusted for age, sex, concomitant pancreatitis, tumor location, tumor differentiation, and interval between initial imaging and operation

*HR* hazard ratio, *CI* confidence interval

* *p*-value less than 0.05

We performed univariate and multivariate Cox regression analyses for overall survival with the same variables used in the Cox regression analyses for recurrence-free survival. In univariate analysis, postoperative CA 19–9 level was the only significant variable (*p* = 0.05). There were no significant variables for overall survival in multivariate analysis (Supplementary Table 1).

## Discussion

This study revealed that a tumor conspicuity score ≥ 2, which represents high lesion contrast on contrast-enhanced CT, significantly correlated with postoperative recurrence in patients with surgically resected pancreatic cancer. In other words, higher lesion contrast or increased tumor conspicuity of pancreatic tumors can be a poor prognostic factor and is related to postoperative recurrence. Conspicuity score was also positively correlated with both radiologic and pathologic pancreatic tumor sizes, and the tumor sizes were significantly larger in the high lesion contrast (conspicuity score ≥ 2) group than for those in the low lesion contrast group (conspicuity score < 2). This result corresponds to a previous report on small PDAC by Yoon et al. [12]. Considering both our study and the Yoon et al. study, smaller tumors tend to present with less prominent tumor conspicuity, and this poor tumor conspicuity changes to increased tumor conspicuity as tumor size increased during the follow-up period. In our study, when comparing initial and preoperative images in 8 patients who underwent delayed surgery more than 1 month after initial imaging, not only tumor size, but also tumor conspicuity were increased, in agreement with the study by Yoon et al.

Several studies have reported that isoattenuating pancreatic tumors show better postsurgical survival compared with conventional pancreatic tumors, although these tumors should not be regarded as early-stage cancers since less than one-third of them are stage T1 [8, 9, 12]. Our study investigated the relationship between poor tumor conspicuity including isoattenuating tumors and patient outcomes, and the results are well-correlated with previous studies. Increased tumor conspicuity (conspicuity score ≥ 2) was a significantly poor prognostic factor for postoperative recurrence in our study but not for overall survival, which is different from the study by Kim et al. [8] . However, the interpretation of survival data for overall survival is limited due to the small number of deaths (*n* = 7) and large number of losses to follow-up. Therefore, survival analysis for overall survival should be further evaluated with a larger study population with long-term follow-up.

Additionally, we performed subgroup analysis according to pathologic tumor size. Both small (size< 3.2 cm) and large (size≥3.2 cm) tumor subgroups in our study showed decreased recurrence-free survival in high-lesion-contrast tumors (conspicuity score ≥ 2) compared with those of low lesion contrast (conspicuity score < 2)(*p* = 0.04), although the small tumor subgroup showed marginally significant results (*p* = 0.06, Supplementary Figure 1). Indeed, larger tumors tended to show increased tumor conspicuity, while small hypoattenuated tumors might also show worse outcomes compared to small isoattenuated tumors within a small tumor group. There is lack of sufficient data comparing small isoattenuating and small hypoattenuating tumors, indicating need for further studies investigating this topic.

The factors of preoperative and postoperative CA19–9 levels, pathologic tumor size, and lymphatic invasion, as well as tumor conspicuity were significant for recurrence-free survival in this study. Apart from pathologic tumor size, neither preoperative nor postoperative CA 19–9 levels showed significant correlation with tumor conspicuity. Preoperative and postoperative CA19–9 levels are well-known prognostic factors for postoperative recurrence and survival [13–16]. We set the median value of 133.1 U/mL of preoperative CA 19–9 and 34.4 U/mL of postoperative CA 19–9 as cut-offs for dividing patients into two groups and compared the outcomes between groups. Higher than median value patients had worse recurrence-free survival for both preoperative and postoperative CA 19–9 (not shown). However, there is controversy about the appropriate cut-off value of CA 19–9 to determine postsurgical outcomes.

Tumor grade can also be a significant prognostic factor for survival [6, 17, 18]; however, in this study, tumor grade had no impact on prognosis. This might be because the majority of the study population had well or moderately differentiated cancers (*n* = 51), rendering little variability within the three grades of tumor. Therefore, we designated ‘tumor grade’ as an adjusting factor for multivariate analysis to yield more accurate results.

The postoperative recurrence rate was quite high (64.5%) in this study despite being lower than in previous studies [13, 15, 19], which were 76.7–92.5%. The recurrence rate in this study could possibly be underestimated due to cases lost to follow-up (*n* = 22) and a short follow-up time within 6 months in some cases. Although eight patients underwent delayed surgery more than 1 month after initial CT imaging, only three patients underwent surgery more than 5 months after initial imaging (6,7, and 20 months), and the interval between initial imaging and surgery for five patients was about 2–3 months. We evaluated postsurgical outcome in the subgroup after excluding eight patients who underwent delayed surgery and confirmed similar results to those of the whole study population (Table 5). We believe there is little impact of delayed surgery on the postsurgical outcomes in this study population because only a small number of patients was affected by a not very long period of delay. The conspicuity scores for tumors in these 8 patients were calculated on the most recently obtained preoperative CT, just prior to surgery, so as to minimize the effect of delayed surgery.

As early recurrence (< 12 months) and distant metastasis (mostly liver metastasis or/and peritoneal seeding) developed in the majority of postoperative recurrences, microscopic metastatic foci, present at the time of resection, could be the causative factor. A more aggressive treatment strategy including neoadjuvant or adjuvant chemotherapy could be recommended in patients with significant predictors for recurrence such as high lesion contrast (conspicuity score ≥ 2), larger tumor size, higher CA 19–9, or presence of lymphatic invasion.

There are several limitations in this study. First, the retrospective design has inherent bias. Second, the small number of study patients limits the comparability of several variables. Third, we developed the new concept of measuring tumor conspicuity as a conspicuity score of P/T AR, which has not been standardized or validated. However, we referred to previous studies when measuring lesion conspicuity [20] and drawing ROIs [12, 21–23] to determine an objective and accurate method for image analysis. Fourth, the dynamic-enhanced CT protocols were heterogeneous, including two-phase and three-phase CTs, owing to the long study collection period. Further, suboptimal CT protocols were used, other than those recommended in Schuller et al. Fifth, there was considerable follow-up loss, and we used the outcomes up through the last visit of those patients. Sixth, this is a single-center study and needs to be confirmed in multicenter investigations and with a larger patient population.

## Conclusion

Tumor conspicuity positively correlates with tumor size both radiologically and pathologically and is also significantly correlated with postoperative recurrence, along with serum CA 19–9 level, tumor size, and pathologic lymphatic invasion, in patients with surgically resected pancreatic cancer. Tumor conspicuity should be considered when planning treatment strategy in patients with tumors that are highly vulnerable to recurrence.

## Supplementary information

**Additional file 1: Table S1.** Cox proportional regression analysis for overall survival (*n* = 62). **Figure S1**. Subgroup analysis according to pathologic tumor size. Both small (A, size< 3.2 cm) and large(B, size≥3.2 cm) subgroups show high lesion contrast tumors with conspicuity score ≥ 2 show decreased recurrence-free survival compared with that of low lesion contrast tumors with conspicuity score < 2 (A, *p* = 0.06, B, *p* = 0.04).

## Data Availability

Not applicable.

## Abbreviations

PDAC: Pancreatic ductal adenocarcinoma
CT: Computed tomography
MRI: Magnetic resonance imaging
CA19–9: Carbohydrate antigen 19–9
TNM: Tumor node metastasis
P/T SIR: Pancreas/tumor signal intensity ratio
ROI: Region of interest
ADC: Apparent diffusion coefficient
HR: Hazard ratio
CI: Confidence interval

## References

[1] Lombardi P, Silvestri S, Marino D (2019). "shades of gray" in pancreatic ductal adenocarcinoma: reappraisals on resectability criteria: debated indications for surgery in pancreatic cancer. Crit Rev Oncol Hematol.

[2] Fitzmaurice C, Dicker D, Global Burden of Disease Cancer C (2015). The global burden of Cancer 2013. JAMA Oncol.

[3] Groot VP, Rezaee N, Wu W (2018). Patterns, timing, and predictors of recurrence following Pancreatectomy for pancreatic ductal adenocarcinoma. Ann Surg.

[4] Wolfgang CL, Herman JM, Laheru DA (2013). Recent progress in pancreatic cancer. CA Cancer J Clin.

[5] Rahib L, Smith BD, Aizenberg R (2014). Projecting Cancer incidence and deaths to 2030: the unexpected burden of thyroid, liver, and pancreas cancers in the United States (vol 74, pg 2913, 2014). Cancer Res.

[6] Rochefort MM, Ankeny JS, Kadera BE (2013). Impact of tumor grade on pancreatic cancer prognosis: validation of a novel TNMG staging system. Ann Surg Oncol.

[7] Sahani DV, Shah ZK, Catalano OA, Boland GW, Brugge WR (2008). Radiology of pancreatic adenocarcinoma: current status of imaging. J Gastroenterol Hepatol.

[8] Kim JH, Park SH, Yu ES (2010). Visually isoattenuating pancreatic adenocarcinoma at dynamic-enhanced CT: frequency, clinical and pathologic characteristics, and diagnosis at imaging examinations. Radiology.

[9] Blouhos K, Boulas KA, Tsalis K, Hatzigeorgiadis A (2015). The isoattenuating pancreatic adenocarcinoma: review of the literature and critical analysis. Surg Oncol.

[10] Tempero MA, Malafa MP, Al-Hawary M (2017). Pancreatic adenocarcinoma, version 2.2017, NCCN clinical practice guidelines in oncology. J Natl Compr Cancer Netw.

[11] Al-Hawary MM, Francis IR, Chari ST (2014). Pancreatic ductal adenocarcinoma radiology reporting template: consensus statement of the Society of Abdominal Radiology and the American pancreatic association. Radiology.

[12] Yoon SH, Lee JM, Cho JY (2011). Small (</= 20 mm) pancreatic adenocarcinomas: analysis of enhancement patterns and secondary signs with multiphasic multidetector CT. Radiology.

[13] Groot VP, Gemenetzis G, Blair AB (2019). Defining and predicting early recurrence in 957 patients with resected pancreatic ductal adenocarcinoma. Ann Surg.

[14] Khorana AA, Mangu PB, Berlin J (2016). Potentially curable pancreatic Cancer: American Society of Clinical Oncology clinical practice guideline. J Clin Oncol.

[15] Kim TH, Han SS, Park SJ (2011). CA 19-9 level as indicator of early distant metastasis and therapeutic selection in resected pancreatic cancer. Int J Radiat Oncol Biol Phys.

[16] Sugiura T, Uesaka K, Kanemoto H (2012). Serum CA19-9 is a significant predictor among preoperative parameters for early recurrence after resection of pancreatic adenocarcinoma. J Gastrointest Surg.

[17] Li J, Liu L (2019). Overall survival in patients over 40 years old with surgically resected pancreatic carcinoma: a SEER-based nomogram analysis. BMC Cancer.

[18] Wu H, Guo JC, Yang SH, Tien YW, Kuo SH. Postoperative Imaging and Tumor Marker Surveillance in Resected Pancreatic Cancer. J Clin Med. 2019;8(8). 10.3390/jcm8081115. 10.3390/jcm8081115 PMC6722558 31357636

[19] Kim NH, Kim HJ. Preoperative risk factors for early recurrence in patients with resectable pancreatic ductal adenocarcinoma after curative intent surgical resection. Hepatobiliary Pancreatic Dis Int. 2018;17(5):450–5. 10.1016/j.hbpd.2018.09.003 30237091

[20] Flechsig P, Zechmann CM, Schreiweis J, et al. Qualitative and quantitative image analysis of CT and MR imaging in patients with neuroendocrine liver metastases in comparison to (68) Ga-DOTATOC PET. Eur J Radiol. 2015;84(8):1593–600. 10.1016/j.ejrad.2015.04.009 25999064

[21] McNamara MM, Little MD, Alexander LF (2015). Multireader evaluation of lesion conspicuity in small pancreatic adenocarcinomas: complimentary value of iodine material density and low keV simulated monoenergetic images using multiphasic rapid kVp-switching dual energy CT. Abdom Imaging.

[22] Schueller G, Schima W, Schueller-Weidekamm C (2006). Multidetector CT of pancreas: effects of contrast material flow rate and individualized scan delay on enhancement of pancreas and tumor contrast. Radiology.

[23] Garces-Descovich A, Morrison TC, Beker K (2018). DWI of pancreatic ductal adenocarcinoma: a pilot study to estimate the correlation with metastatic disease potential and overall survival. Am J Roentgenol.

