# Supplementary material for: Tumor conspicuity significantly correlates with postoperative recurrence in patients with pancreatic cancer: a retrospective observational study
Source: Cancer Imaging. 2020 Jul 10;20:46. doi: 10.1186/s40644-020-00321-2 (PMC7350737; doi:10.1186/s40644-020-00321-2)

Supplementary Table 1. Cox proportional regression analysis for overall survival (n=62)

|  | Univariate:  HR (95% CI) | p-value | ^§^Multivariate:  HR (95% CI) | p-value |
| --- | --- | --- | --- | --- |
| Charlson age-comorbidity  Index | 1.084(0.683-1.720) | 0.732 |  |  |
| Interval from initial imaging  And operation (mo.) | 0.994(0.787-1.254) | 0.958 |  |  |
| Adjuvant chemotherapy | 1.958(0.233-16.435) | 0.536 |  |  |
| Preoperative CA 19-9 | 1.068(0.886-1.287) | 0.491 | 0.964(0.711-1.305) | 0.81 |
| Postoperative CA 19-9 | 1.15(1.000-1.322) | 0.05* | 1.142(0.912-1.431) | 0.247 |
| Radiologic tumor size | 0.584(0.202-1.686) | 0.320 | 0.534(0.13-2.188) | 0.383 |
| Resectability  Resectable  Borderline resectable  Unresectable | 1  0.441(0.051-3.781)  3.965(0.428-36.6890 | 0.455  0.225 |  |  |
| Conspicuity score>2 | 0.726(0.132-3.989) | 0.712 | 0.748(0.106-5.258) | 0.77 |
| Pathologic tumor size | 0.831(0.402-1.719) | 0.618 | 0.49(0.163-1.478) | 0.206 |
| Positive resection margin | 2.056(0.224-18.834) | 0.524 |  |  |
| Lymphatic invasion | 1.49(0.261-8.522) | 0.654 | 2.085(0.187-23.28) | 0.551 |
| Perineural invasion | 2.707(0.486-15.074) | 0.256 | 3.828(0.543-27.001) | 0.178 |
| Microvascular invasion | 2.791(0.459-16.966) | 0.265 | 4.418(0.482-40.483) | 0.189 |
| Advanced pathologic T stage  (T3,T4) | 1.586(0.187-13.455) | 0.672 | 0.835(0.079-8.875) | 0.881 |
| Positive lymph node | 1.062(0.231-4.870) | 0.939 | 2.051(0.254-16.556) | 0.5 |
| Postoperative recurrence | 40.532(0.05-32088.51) | 0.277 |  |  |

§ Adjusted for age, sex, concomitant pancreatitis, tumor location, tumor differentiation and interval between initial imaging and operation

HR, hazard ratio; CI, confidence interval

Supplementary Figure 1. Subgroup analysis according to pathologic tumor size. Both small (A, size<3.2 cm) and large(B, size≥ 3.2 cm) subgroups show high lesion contrast tumors with conspicuity score≥2 show decreased recurrence-free survival compared with that of low lesion contrast tumors with conspicuity score<2 (A, p=0.06, B, p=0.04).


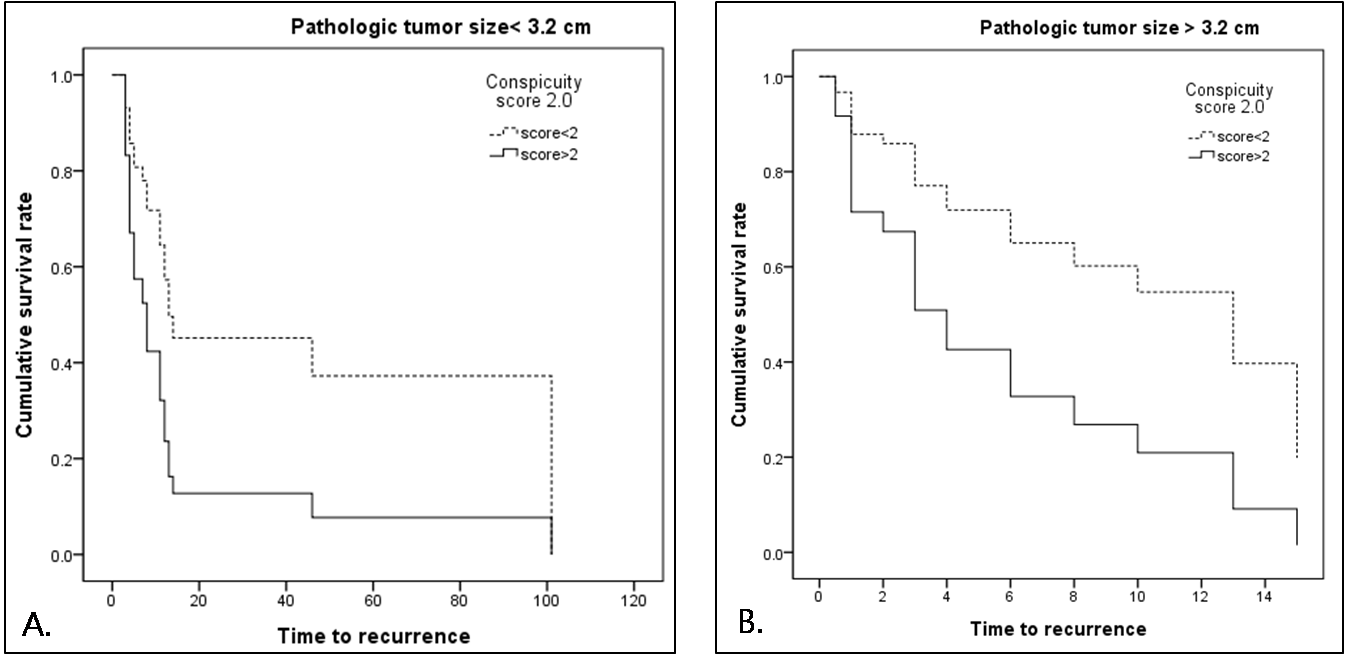

Supplement: Supplementary file 1 — Additional file 1: Table S1. Cox proportional regression analysis for overall survival (n = 62). Figure S1. Subgroup analysis according to pathologic tumor size. Both small (A, size< 3.2 cm) and large(B, size≥3.2 cm) subgroups show high lesion contrast tumors with conspicuity score ≥ 2 show decreased recurrence-free survival compared with that of low lesion contrast tumors with conspicuity score < 2 (A, p = 0.06, B, p = 0.04). [file 40644_2020_321_MOESM1_ESM.docx]
